# Supplementary material for: Hunting regulation favors slow life histories in a large carnivore
Source: Nat Commun. 2018 Mar 27;9:1100. doi: 10.1038/s41467-018-03506-3 (PMC5871616; doi:10.1038/s41467-018-03506-3)
Supplement: Supplementary file 1 — Supplementary Information(PDF 491 kb) [file 41467_2018_3506_MOESM1_ESM.pdf]

Hunting regulation favors slow life histories in a large carnivore

Van de Walle et al.

Supplementary Information

Supplementary Table 1 | Empirical demographic rates of brown bear females in south-central Sweden from 1993-2015

	1.5-year tactic			2.5-year tactic			All females*		
Demographic rate	Mean	SE	n	Mean	SE	N	Mean	SE	n
S_1	0.780	0.038	118	1.000	0.000	32	0.827	0.031	150
S_2	0.860	0.037	86	0.862	0.064	29	0.866	0.031	119
S_3	0.931	0.030	72	0.895	0.070	19	0.919	0.027	99
$S_{4\text{-}8}$	0.973	0.013	149	0.985	0.015	65	0.911	0.015	370
S_{9+}	0.841	0.027	182	0.916	0.027	107	0.831	0.020	355
$R_{5\text{-}9}$	0.327	0.051	147	0.297	0.091	64	0.241	0.035	278
R_{10+}	0.471	0.068	140	0.233	0.057	90	0.367	0.047	237
λ†	1.090 [1.052, 1.127]			1.101 [1.040, 1.165]			1.031 [1.000, 1.062]		

Observed mean, standard error (SE), and sample size (n) of age-specific survival probability (*S*) and recruitment rate (*R* = number of female yearlings produced per female per year) along with resulting asymptotic population growth rate (λ) for the two maternal care tactics. Definitions: S_1 = survival of yearlings, S_2 = survival of 2 year-olds (y.o.), S_3 = survival of 3 y.o., $S_{4\text{-}8}$ = survival of 4-8 y.o., S_{9+} = survival of 9 y.o. and older, $R_{5\text{-}9}$ = recruitment of 5-9 y.o. and R_{10+} = recruitment of 10 y.o. and older. * For comparison, information is also provided for the entire population when considering all females within the population. This includes females that were excluded from the tactic-based models because we were unable to classify them within one tactic or the other due to, for example, an impossibility to determine weaning time.

† λ here was calculated using observed demographic rates

Supplementary Table 2 | Estimates (β and 95% CI) of the effect of hunting pressure on the survival of female brown bears using either the 1.5-year or the 2.5-year maternal care tactics in south-central Sweden, 1993-2015

	1.5-year tactic			2.5-year tactic		
Age-class	Mean	Lower	Upper	Mean	Lower	Upper
Yearling	-0.362	-5.552	4.993	-	-	-
2 y.o.	-8.264	-18.736	-0.087	-10.311	-32.032	7.549
3 y.o.	-11.176	-28.692	2.450	-12.517	-47.580	13.195
4-8 y.o.	-17.104	-41.043	-0.761	3.187	-25.622	30.478
9+ y.o.	-6.312	-12.617	-0.625	-2.907	-15.266	6.860

Posterior means of estimated effect sizes (log-odds) are given along with their 95% highest posterior density intervals. These effect sizes were used to make model predictions for a range of hunting pressures observed in the population between 1993-2015. For each hunting pressure simulated, predictions of survival probabilities were then back-transformed on the original scale and incorporated into hunting pressure-specific Leslie projection matrix models for each tactic to extract the asymptotic population growth rate.

Supplementary Table 3 | Estimates (β and 95% CI) of the effect of population density on the survival probability of female brown bears using either the 1.5-year or the 2.5-year maternal care tactics in south-central Sweden, 1998-2015

	1.5-year tactic			2.5-year tactic		
Age-class	Mean	Lower	Upper	Mean	Lower	Upper
Yearling	1.618	-0.2944	3.450	-	-	-
2 y.o.	0.522	-2.373	3.577	-9.448	-23.923	2.595
3 y.o.	0.562	-3.869	4.369	-0.008	-5.330	4.809
4-8 y.o.	1.712	-2.115	5.170	4.536	-4.782	14.139
9+ y.o.	-1.726	-3.536	0.030	-1.758	-6.260	2.928

Posterior means of estimated effect sizes (log-odds) are given along with their 95% highest posterior density intervals. These effect sizes were used to make model predictions for a range of population densities observed in the population between 1998-2015. For each population density simulated, predictions of survival probabilities were then back-transformed on the original scale and incorporated into population density-specific Leslie projection matrix models for each tactic to extract the asymptotic population growth rate.

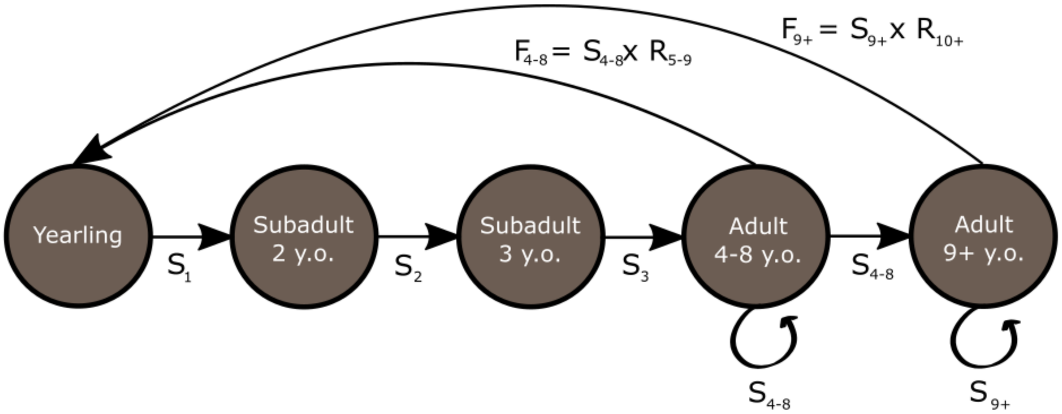

Supplementary Figure 1 | Life cycle graph of a female brown bear in Scandinavia. The life cycle graph was used to construct age-structured population models. Definitions: S_1 = survival of yearlings, S_2 = survival of 2 y.o. (y.o. = years old), S_3 = survival of 3 y.o., S_{4-8} = survival of 4-8 y.o., S_{9+} = survival of 9 y.o. and older, S_{4-8} = fecundity of 4-8 y.o., F_{9+} = fecundity of 9 y.o. and older, R_{5-9} = recruitment of 5-9 y.o. and R_{10+} = recruitment of 10 y.o. and older.

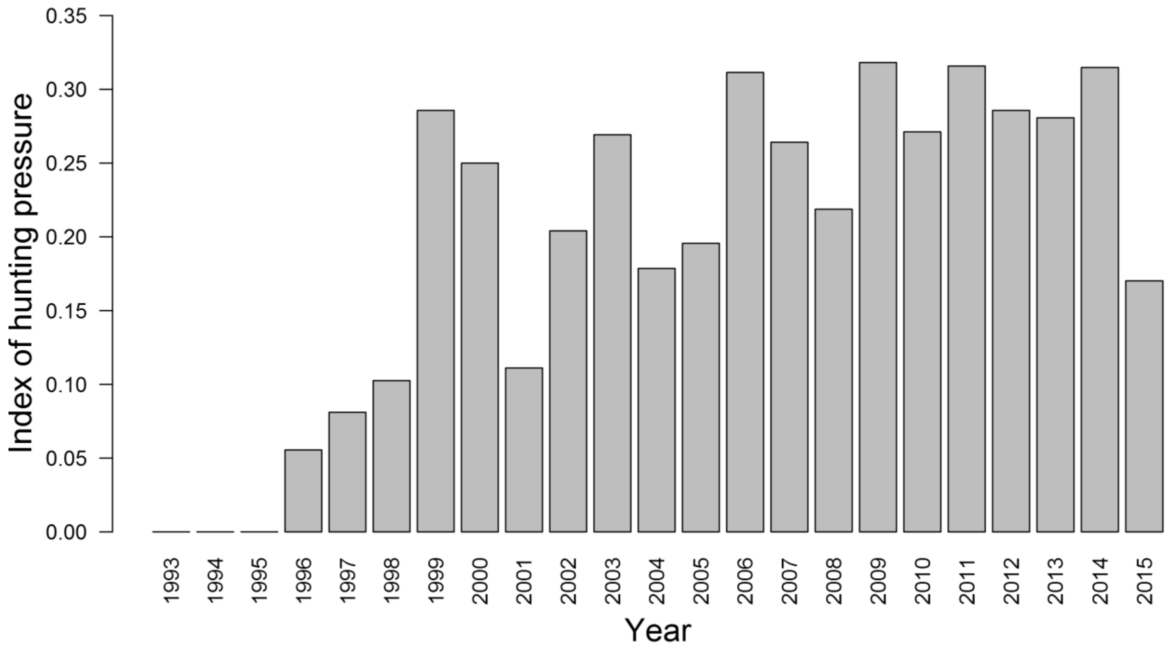

Supplementary Figure 2 | Annual index of bear hunting pressure in south-central Sweden from 1993-2015. The index was estimated using all marked brown bears, i.e., by dividing the number of marked bears that were shot in a given year by the number of marked bears available for hunting that same year.

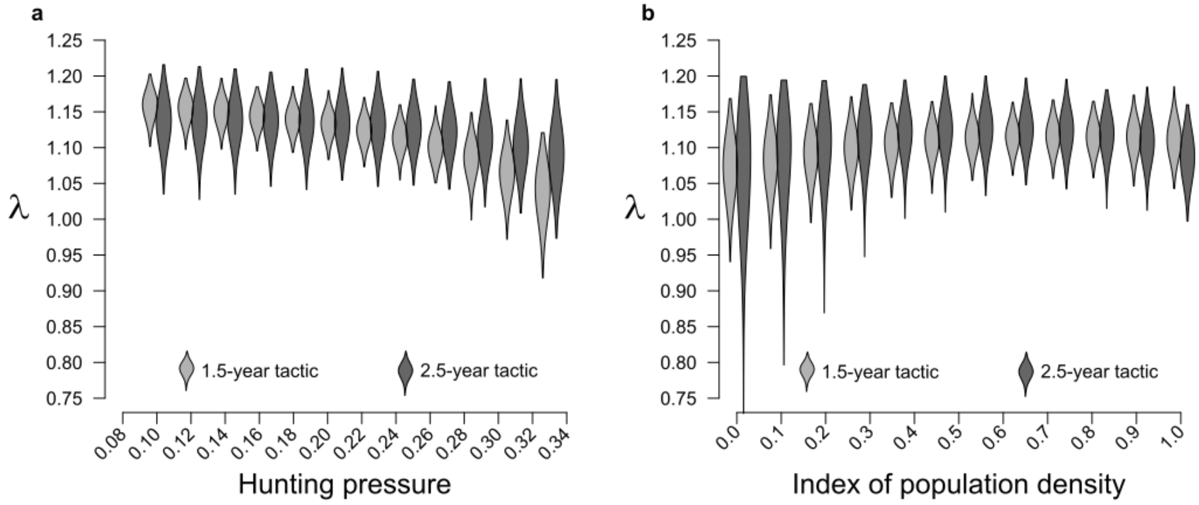

Supplementary Figure 3 | Effect of hunting pressure and population density on the fitness of female brown bears using either the 1.5-year or the 2.5-year maternal care tactic in south-central Sweden from 1998-2015. Violin plots representing the predicted effects of **a** hunting pressure and **b** the index of population density on asymptotic population growth rate, λ, for each maternal care tactic. The violins represent the density plots of lambda (1,000 iterations) at each hunting pressure and population density index simulated. Because density could be estimated only for 1998-2015, the effect of hunting pressure over the same period is presented to provide adequate comparison. The effect of hunting pressure on tactic fitness considering the entire study period (1993-2015) is presented on Fig. 5.
